# Physical activity among South Asian women: a systematic, mixed-methods review

**DOI:** 10.1186/1479-5868-9-150

**Published:** 2012-12-20

**Authors:** Whitney S Babakus, Janice L Thompson

**Affiliations:** 1 School of Sport and Exercise Sciences, University of Birmingham, Birmingham, B15 2TT, UK

**Keywords:** Health inequalities, Sedentary time, Self-reported physical activity

## Abstract

**Introduction:**

The objective of this systematic mixed-methods review is to assess what is currently known about the levels of physical activity (PA) and sedentary time (ST) and to contextualize these behaviors among South Asian women with an immigrant background.

**Methods:**

A systematic search of the literature was conducted using combinations of the key words PA, ST, South Asian, and immigrant. A mixed-methods approach was used to analyze and synthesize all evidence, both quantitative and qualitative. Twenty-six quantitative and twelve qualitative studies were identified as meeting the inclusion criteria.

**Results:**

Studies quantifying PA and ST among South Asian women showed low levels of PA compared with South Asian men and with white European comparison populations. However making valid comparisons between studies was challenging due to a lack of standardized PA measurement. The majority of studies indicated that South Asian women did not meet recommended amounts of PA for health benefits. Few studies assessed ST. Themes emerging from qualitative studies included cultural and structural barriers to PA, faith and education as facilitators, and a lack of understanding of the recommended amounts of PA and its benefits among South Asian women.

**Conclusions:**

Quantitative and qualitative evidence indicate that South Asian women do not perform the recommended level of PA for health benefits. Both types of studies suffer from limitations due to methods of data collection. More research should be dedicated to standardizing objective PA measurement and to understanding how to utilize the resources of the individuals and communities to increase PA levels and overall health of South Asian women.

## Background

Low levels of physical activity (PA) (defined as movement of the body requiring energy expenditure) and increased sedentary time (ST) (no energy expenditure above that required at rest) are major independent risk factors in the development of cardiovascular disease, and are recognized as key contributors to other chronic conditions such as type 2 diabetes and obesity [1]. As such, PA and ST are potentially modifiable health behaviors that can be changed to reduce risks for morbidity and premature mortality resulting from various chronic diseases [2-4]. It has been shown that those who are physically active can reduce their risk of developing cardiovascular disease by up to 50% [2]. The World Health Organization, US Department of Health and Human Services and the UK Department of Health agree that 150 minutes of moderate intensity PA or 75 minutes of vigorous activity per week are recommended to achieve health benefits [5-7].

South Asian women (SA) are disproportionately affected by chronic diseases [2,8,9] and large cross-national surveys of many western nations such as the UK, Australia and the US have consistently reported that SAs report lower levels of PA than their white counterparts [10,11]. Cardiovascular disease (CVD), which includes heart attack and stroke, accounts for 16.7 million deaths globally each year [12]. CVD is the leading cause of death in the UK and nearly half of all deaths in Europe are caused by CVD [12]. The UK has one of the highest rates of CVD in the world, with more than 190,000 deaths in 2008 [12]. In the UK 33% of all mortality in South Asian women and 25% in men 2008 was caused by cardiovascular disease [12]. As insufficient PA is recognized as a significant, independent risk factor for CVD and other chronic diseases, it is important to gain a better understanding of the PA levels of SA and the factors influencing PA among this population to help inform the development and delivery of culturally appropriate interventions and policy [13].

Researchers have recently focused on quantifying and contextualizing PA and ST among groups such as SAs living in industrialized countries who are at the greatest risk for cardiovascular and other chronic diseases such as type 2 diabetes and hypercholesterolemia when compared to the general populations of those countries [14]. The objective of this systematic mixed-methods review is to assess what is currently known about levels of PA and ST in SA. Research questions are: 1) What is known about the volume, intensity, duration and type of PA/ST that SAs engage in, 2) What is known about the context in which these behaviors occur in SA, and 3) What is the quality of the evidence on PA/ST in SAs?

## Methods

After careful consideration of the methods that would be most appropriate for answering the research questions, a combination of the EPPI-Centre mixed-methods systematic review and an integrative review were used. This review uses the EPPI-Centre methods for systematically searching the literature, rigorously assessing the quality of studies, and synthesizing quantitative and qualitative studies into one report. As in the integrative method, many types of studies were considered for inclusion in order to meet the objective of the review [15]. These included randomized and non-randomized controlled trials, observational studies and qualitative studies. The adaptation of the mixed-methods review and integrative review allowed for a systematized and rigorous review while including the appropriate studies to fully answer the research questions.

### Search strategy

A search was performed in online databases (MEDLINE, *The Cochrane Library*, EMBASE, PsychInfo, CINAHL, AnthroSource and Sociological Abstracts from 1980 to July 2012), grey literature, hand searches of journals over the previous twelve months (*MSSE, Journal of Physical Activity & Health, Journal of Aging and Physical Activity)*, and reference lists of articles to identify studies. Search terms included combinations of PA, ST, physical exercise, physical fitness, exercise, sport, physical training, physical training, recreation activity, MVPA, LTPA, leisure activities, physical inactivity, sedentary behavior and South Asian/immigrant. Inclusion criteria were: Randomized and non-randomized controlled studies, observational and qualitative studies; studies that include data on PA and/ or ST; studies on SA; studies published from 1980 on to obtain the most current data; studies with data on adult women aged 18 and older; studies published in English. Exclusion criteria included: Studies without adult data and studies focusing on migrant groups instead of permanent immigrants, and studies on children.

Quality of studies was assessed using validated checklists developed from the Critical Appraisal Skills Programme (CASP) [16,17]. The checklist for quantitative studies was used to assess quality of study design including methods selection, identification of biases, appropriate use of statistical methods, and clarity of reporting [16]. Studies were accepted for inclusion if they addressed each of these through justification of choices made in each quality category. The checklist for qualitative studies assessed strength of studies for inclusion, ranking studies on a continuum from weak to strong [17]. Consideration was given to rigor (thorough and appropriate key research methods), credibility (findings well presented and meaningful), and relevance (how useful are the findings) [17]. Qualitative studies were acceptable for inclusion if they were categorized as at least of moderate quality. Study eligibility was confirmed by two researchers and quality assessment and data extraction were performed by the principal investigator (WSB) and confirmed by a senior researcher (JLT).

Findings from quantitative studies are described numerically and textually to provide a summary of evidence on PA and ST performed by SA since heterogeneity of methods and measures of PA and ST precludes the use of meta-analysis. Qualitative data was synthesized and analysed thematically using NVIVO in three stages: 1) line-by-line coding of primary studies; 2) organising codes into themes; and 3) development of analytical themes [18]. The final integrated synthesis consists of narrative commentary, combining the results of quantitative and qualitative syntheses.

## Results

**Figure 1:**
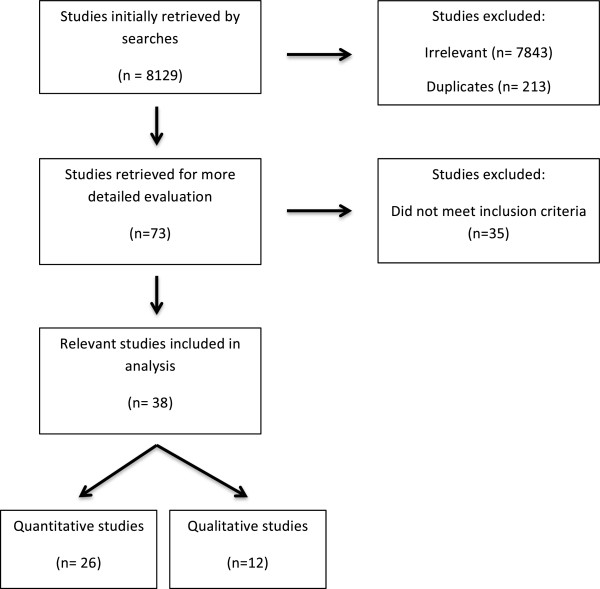
Identification and inclusion of relevant studies for review

Figure 1 shows the identification and inclusion of studies for the final synthesis. A total of 8,123 studies were initially retrieved and seventy-three studies were identified by title and abstract for more detailed evaluation. Thirty-eight studies (quantitative n = 26, qualitative n = 12) were included in the final synthesis and analysis.

### Quantitative synthesis

One study was observational longitudinal [4] and 25 were cross-sectional designs [7,8,10,19-39] (Table 1). Fifteen studies were conducted in the UK [4,10,19,20,24,26,32-34,36-38,40-42], six in the US [21,27-29,31,39], two in Canada [8,30], one in New Zealand [22], one in Australia/India [25], and one in Guadeloupe [35]. Eight studies obtained samples from large-scale population studies [10,29,30,33,36-39], five recruited from community centres [21,22,27,28,32], five from census/birth records or electoral registers [20,34,40-42], three from general practice lists [11,26,35], two did not state recruitment strategy [8,43], two recruited based on postcode [4,19] and one recruited from a university campus [31]. Five studies were limited to women [20,24,31,33,40], while the remaining included both women and men. Five studies conducted their analyses on men and women as one group [10,21,29,36]; sixteen provided analyses by gender [4,8,19,22,25-27,30,32,33,35,37,38,41-43]. Four studies conducted subgroup analyses by country of origin [4,10,19,20]. All but two [22,32] studies assessed PA or ST through self-report survey. These were through pedometer (New Lifestyles NL200) to calculate steps and through accelerometer (*Caltrac*) used in conjunction with a heart rate monitor to calculate energy expenditure [22,32]. Four studies [21,22,26,33] examined PA among SA over 40 years of age while all other studies examined adults ranging in age from 16 yr to 90+ yr.

**Table 1:** Summary of included quantitative studies

**+**	**Location of fieldwork**	**Participants & sample recruitment**	**Design**	**Physical activity measures**	**Translation**	**Response rate**	**Main findings**
Dogra et al., 2010	Canada	N = 347,229	Cross-sectional	Self-report: 3 month recall of PA (metabolic equivalent calculation based on Canadian Fitness and Lifestyle Research Institute cut-offs	NA**	NA	SA less likely than Whites to engage in walking, endurance, recreation, and sport activities (SA: walking = 56.7%, endurance = 29.7%, recreation = 38.3%, sports = 24.3%; Whites: walking = 68.3%, endurance = 34.6%, recreation = 60.0%, sports = 28.8%). SA report more inactivity: 18.7%, Whites = 10.5%
		N(male White) = 10729					
		N(female White) = 114,965					
		N(male SA*) = 1,708					
		N(female SA) = 1,576					
Health Education Authority, 2000	UK	N = 4,444	Cross-sectional	Self-report: survey piloted and revised for clarity	Translated into 7 languages	72%	% reporting taking ‘regular exercise:
		N(Indian) = 1,111					Indian = 71%
		N(Pakistani) = 1,111					Pakistani = 63%
		N(Bangladeshi = 1,111					Bangladeshi = 65%
		N(Afro-Caribbean) = 1,111					% females reporting ‘very active’:
		National Survey of Ethnic Minorities					Indian = 17%
							Pakistani = 18%
							Bangladeshi = 17%
Hine et al., 1995	UK	N = 547 (women only)	Cross-sectional	Self-report	Translated into 7 languages	71%	% currently doing exercise to keep healthy:
		N(Pakistani) = 79					Pakistani: 1%, Indian: 6%, Bangladeshi: 12.5%
		N(Indian) = 52					
		N(Bangladeshi) = 21					
		Identified from Family Health Services Authority and Electoral Register					
Jonnalagadda & Diwan, 2002	US	N = 237 Asian Indian men and women	Cross-sectional	Self-report: survey based on Kriska et al, 1997	NA	65%	% reporting engaging in 1 or more of the 3 activities from PA index:
		Identified from 10 community organizations					South Indian = 70%
							North Indian = 56%
							West Indian = 65%
Kolt et al., 2007	New Zealand	N = 112	Cross-sectional	Objective measurement: New Lifestyles NL2000 pedometer	NA	NA	48% of total sample classified at sedentary (<5000 steps/day)
		N(Asian Indian men) = 50 N(Asian Indian women) = 62					33% classified as active (>10,000 steps/day)
		Identified from Auckland-based Asian Indian community organizations					
Lean et al., 2001	UK	N = 259	Cross-sectional	Self-report	NA	76%	18% of Migrant SA performed sport and exercise
		N(Scotland general population) = 50					30% of British-born SA performed sport and exercise
		N(immigrant SA ) = 63					
		N(UK-born SA ) = 56					
		N(immigrant Italians ) = 39					
		N(UK-born Italians ) = 51					
Lip et al., 1996	UK	N = 232 (women only)	Cross-sectional	Self-report of regular exercise	Translated into 3 languages	NA	Lower proportion of exercisers among South Asians (*X*2 = 22.34, df = 2, p < 0.001)
		N(White ) = 84					
		N(SA) = 72					
		N(Afro-Caribbean ) = 76					
		Recruited from City Hospital, Birmingham					
Mahajan & Bermingham, 2004	Australia/India	N = 250	Crosssectional	Self-report: Based on the National Heart Foundation Risk Factor Survey	NA	63%	Total exercise hours/week: Men in Australia:17.3+/-25.5
		N(SA Indians in Australia ) = 125					Men in India: 18.9+/-29.4 Women in Australia: 17.1+/-20.6
		N(familial relatives in India ) = 125					Women in India: 33.5+/-36.9 (P < 0.001 referring to country of residence stratified by gender)
		Recruited from Indian community centres in Sydney and referred familial relatives in India					
McKeigue et al., 1992	UK	N = 3,399	Cross-sectional	Self-report	Completed questionnaire checked by bilingual fieldworker	NA	Age-adjusted mean leisure time:
		N(European men) = 1,506					SA: 3.0 MJ/week
		N(SA men) = 1,360					European: 4.2 MJ/week P < 0.001
		N(European women) = 245					
		N(SA women) = 288					
		Recruited from general practitioner’slists and industrial workforces in West London					
Misra et al., 2005	US	N = 56 SA Indian immigrants	Cross-sectional	Self-report: Minnesota LTPA*** questionnaire	NA	80%	Total activity mean in min/week
		N = 31 men					Men: 124.5+/-107.8
		N = 25 women					Women: 50.2+/-62.3
		Recruited via general practitioner’s offices, community centres and media releases					
Misra, 2004	US	N = 261 Gujarati Asian Indian immigrants	Cross-sectional	Self-report: revised Health Promotion Lifestyle Profile II	NA		53.3% Follow exercise regime
		N(men ) = 180					56.4.% of men
		N(women ) = 81					52.5% of women
							Significant difference between men and women (*X*2 = 14.1, p = 0.001)
Mohanty et al., 2005	US	N = (White) 87,846	Cross-sectional	Self-report: any vigorous activity 10-20 min at least once per week	NA	80.4% in1997, 73.9% in 1998, 69.6% in 1999, 72.1% in 2000	% reporting never being active or active less than once/week:
		N(SA Indian) = 555					White = 59.3%
		National Health Interview Survey years 1997-2000					Asian Indian = 67% (p = .004)
O’Laughlin et al., 2007	Canada	N = 2033 (42.2% male)	Cross-sectional	Self-report: ≥20 min. LTPA at least twice/week for 4 months	NA	NA	% inactive (95%Confidence Interval)
		N(French Canadian) = 575					French Canadian = 71.5% (67.6-75.1)
		N(Portuguese) = 294					Portuguese = 80.5%(75.5-84.9)
		N(Italian) = 122					Italian = 78.3%(69.9-85.3)
		N(Eastern European) = 51					Eastern European = 58%(43.2-71.8)
		N(SA) = 42					SA = 76.2%(60.6-88.0)
		Data available from adult parents of children participating in an intervention in Montreal					
Palaniappan et al., 2002	US	N = 210	Cross-sectional	Self-report	NA	71.40%	Years of regular exercise Caucasian: 6.2+/-4.0
		N(Caucasian 0 = 67					African American: 4.0+/-4.2 SA Indian: 4.2+/-4.3 P = 0.0013
		N(African American ) = 69					
		N(SA Indian ) = 70					
Patel et al., 2006	UK/India	N = 537 total	Cross-sectional	Objective Measurement: Caltrac accelerometers	Bilingual fieldworkers conducted measurements	67% in Sandwell, 65% in Navsari	Measured physical activity in Kcal/day (95% CI): Men in India:1820(1630-2000)
		N(SA Indian men in UK ) = 119					Men in UK: 2350(2200-2490)
		N(SA Indian men in India) = 139					Women in India: 1680(1540-1810) Women in UK: 1750(1640-1870)
		N(SA Indian women in UK) = 123					
		N(SA Indian women in India) = 155					
		Recruited from community directories and local primary care registries in UK, from electoral roll from India					
Pomerleau et al., 1999	UK	N = 839 (women only)	Cross-sectional	Self-report	Bilingual fieldworkerscollected data and translated during interview	NA	SA women walked least for transport compared to European and Afro-Caribbean: 22% vs 44% and 40%, respectively. 1% of SA women participated in sport and none cycled
		N(European ) = 246					
		N(SA) = 291					
		N(Afro-Caribbean ) = 303					
		Data from 2 large cross-sectional studies, Southall and Brent surveys					
Riste et al., 2001	UK	N = 919	Crosssectional	Self-report: validated questionnaire(Was hburn et al, 1990), PA reported over the past week	Punjabi and Urdu interviewers available	65%	% physically active defined as 3X20min/week (95% CI):
		N(European ) = 471					Pakistani men = 6.8%(0-13)
		N(Pakistani ) = 132					Pakistani women = 5.2%(0-11)
		N(Afro-Caribbean ) = 316					European men = 37.8%(23-53)
		Sampled from registers from local health centres					European women = 29.4%(13-46)
Rudat, 1994	UK	N = 2,619	Crosssectional	Self-report		Indian = 77%, Pakistani = 80%, Bangladeshi = 9 1% successful as% of screened eligible respondents	% reporting any activity: Indian = 46%, Pakistani = 41%, Bangladeshi = 37%
		N(SA Indian ) = 1017					
		N(Pakistani ) = 935					
		N(Bangladeshi ) = 667					
		Sample available from 1981 census					
Sinnapah et al., 2009	Guadeloupe	N = 122	Crosssectional	Self-report: 24-hour recall	NA	93%	Energy expenditure in Kcal +/-SD: SA Indian men: 2615+/-417
		N(general population men) = 25					SA women: 2264+/-465
		N(SA Indian men) = 27					Controls men: 2921+/-608
		N(general population women) = 32					Controls women: 2481+/-627
		N(SA women) = 30					
		Sampled from those workers who came in to attend annual medical check-up					
Williams et al., 2010	UK	N = 15,413	Observational longitudinal	Self-report: 4 week recall	Questions translated into 5 languages	69-76%	% reporting no weekly physical activity(unadjusted):
		N(White) = 13,293					White = 28.1%
		N(SA Indian) = 1,244					Indian = 37.1%
		N(Pakistani/Banglade shi ) = 876					Pakistani/Bangladeshi = 56.7% (p < 0.001)
		Data available from Health Survey for England years 1999 and 2004					
Williams et al., 2010a	UK	N = 1,948	Crosssectional	Self-report: Based on IPAQ****	Questionnair e available in English and Punjabi	83%	% reporting more than 3 hours sedentary/day:
		N(White) = 818					SA = 45.6%
		N(SA) = 1130					White = 47.5%
		Recruited from London Life Sciences Prospective Population(LOLIPOP) study					% reporting some physical activity:SA = 73.2%
							White = 79.4%
Williams et al., 1994	UK	N = 173 SA	Crosssectional	Self-report	Bilingual interviewer and questionnaire available in 4 languages	80.5%	% reporting ever taking vigorous exercise: SA Males = 46%, Male general population = 59% SA females = 38%, Female general population = 44% SA less likely to report ever taking vigorous exercise, difference statistically significant in men (p < 0.05)
		N by sex unspecified					
		Sampled from electoral and valuation rolls in Glasgow					
Yates et al., 2010	UK	N = 5,474	Crosssectional	Self-report: Short version of last seven-day self administered format of IPAQ	English only	92% of white European, 69% of SA	% in activity level category:
		N(White men) = 2033					White Men: Low = 22%, Moderate = 28%, High = 50%
		N(SA men) = 604					SA Men: Low = 37%, Moderate = 25%, High = 38%
		N(White women) = 2277					White Women: Low = 27%, Moderate = 33%, High = 32%
		N(SA women) = 560					SA Women: Low = 40%, Moderate = 28%, High = 32% (all significant at p < 0.01)
		European -baseline data from ADDITION-Leicester study					
Yates et al., 2012	UK	N = 505	Crosssectional	Self-report: Short version of last seven-day self administered format of IPAQ	English only	NA	Total hours sitting time (hours/day):
		N(White European Men) = 220					Men = 6.0(4.0-8.8)
		N(White European Women) = 188					Women = 5.0(4.0-7.0)
		N(South Asian Men) = 52					*p* for difference between genders <0.01
		N(South Asian Women) = 45					Total MVPA + (METhours/week:
		From subsample of the ADDITIONLeicester study from 2004-2007					Men = 46(17-108)
							Women = 34(17-106)
							*p* for difference between genders <0.01
Ye et al., 2009	US	N = 77,267	Cross-sectional	Self-report	NA	NA	% reporting physical inactivity(unadjusted):
		N(White) = 74,424					White = 37.2%, Asian
		N(SA Indian) = 534					Indian = 41.8%, Other
		N(Other Asian) = 1,117					Asian = 41.0% (*X*2 = 16.27, p = 0.04)
		Aggregated data from the National Health Interview Survey 2003 to 2005					

*South Asian, **Information not available in article, *** Leisure time physical activity, ****International Physical Activity Questionnaire, +Moderate-to-vigorous physical activity.

### Measurement of physical activity and sedentary time

Sixteen studies focused on PA prevalence [4,8,10,19,21,22,24,27,29,30,33,34,38-40,42]. Two studies used a questionnaire to estimate both PA and ST among male and female SAs in the UK [36,39]. Fourteen focused on the prevalence of PA among SAs in comparison to the white or general population of the host country. Kolt et al. (2007) did not make this comparison [22]. Methods of assessing PA prevalence varied greatly between studies. Four studies measured prevalence of PA in the week prior to completion of the survey [10,29,34,39], two asked respondents broadly if they participated in PA but did not specify a timeframe [21,40], two assessed the prevalence of participating in regular PA [24,30], one dichotomized PA by asking respondents if they were either active or inactive [4], one assessed prevalence of PA based on the number of kilometres walked or cycled per hour/week [33], one assessed the prevalence of ever taking vigorous PA [42], one used a scale (highly to ‘never-routinely’) [28], one study assessed the prevalence of performing PA by number of times/week [8], one assessed the prevalence of physical inactivity only [39], and one assessed PA using pedometer steps per minute measured over 7 consecutive days [22]. All studies that assessed prevalence of PA in comparison to the white or general population found that SAs performed significantly less PA than the comparison group [4,8,10,24,29,33,39,40,42,44].

Three studies assessed duration of PA [25,27,31]. Misra et al. (2005) report total PA duration in mean minutes per week [27]. SA Indian men performed an average of 45 (+/−44) minutes while women performed 16 (+/−48) minutes of PA, less than half of the minutes of PA that the men did per week [27]. Majahan et al. (2004) reported total exercise in hours over a two-week period [25]. This study compared SA Indian women living in Australia or India, and found that women in Australia were performing just over half of the hrs/2 weeks of PA (4.8 +/5.3, p < .003) than the women in India (9.2+/− 9.1, p < .003) [25]. Palaniappan et al. (2002) reported regular exercise in number of minutes per week [31], and found that SA Indian women performed nearly 5 minutes less regular exercise overall than their white counterparts (42 +/− 20.7 vs. 46.5 +/− 21.5) though this result was not significant. Two studies assessed PA in terms of energy expenditure with both finding that SA expended less energy in PA that their white and SA male counterparts [26,35]. The one study that measured both PA and ST reported that on average, 70% of SAs in the study did some PA and that an average of 50% of participants were sedentary for more than 3 hours per day [36].

Eighteen studies assessed LTPA [8,10,19-21,24-31,33,34,40,41,45], three assessed home, work and leisure physical activity combined [4,37,38], two assessed active commuting [8,27], two did not specify mode of activity but instead measured energy expenditure [32,35], one assessed occupational physical activity [33], one study did not specify mode of activity but focused on intensity of the activity [42], one study assessed steps but did not specify the mode of activity [22], and one study looked solely at physical inactivity with no mode specified [39]. In studies that assessed LTPA in both SA and white European groups, SA consistently reported less LTPA than their white European counterparts [4,8,10,24-26,29-31,33,34,40,41]. In the study by Dogra et al., 55.8% of SA respondents reported actively commuting to work versus 53.1% of the white European respondents (not significant) [8]. Misra et al. (2005) report that 12.5% of SA men reported actively commuting to work while no women reported doing so [27]. Pomerleau et al. (1999) indicate that 62.5% of SA and 49.0% of white Europeans reported walking more than sitting at work (p < 0.01) [33].

### Qualitative synthesis

Eleven of the twelve qualitative studies included in this review investigated LTPA [15,23,46-53], and one investigated the feasibility of using accelerometers and questionnaires to assess PA among SA [54]. Seven conducted individual interviews [22,27,32,46,47,55,56], three conducted focus groups [50,52,57], and two used both methods to address various research questions pertaining to PA; none of these studies explored behaviors related to ST [48,49]. Eight studies were conducted in the UK [23,46,48,49,52-54,58], and one each in Canada [47], Australia [51], the US [50], and Norway [15]. Participants from five studies were recruited from general medical practices [23,46,47,51,52,58], three from community centers/sports clubs [15,52,54], one via media release [51], one recruited participants by identifying people from participant observations [49], one from a larger cross sectional study [50] and one did not describe participant recruitment [48]. Nine studies included male and female participants [23,46-52,58] and three were restricted to women [15,53,54]. Ages of participants in all studies ranged from 30 yr to 70+ yr. One study restricted age to 16–25 yr [15] and one did not specify age, but has been included in this review because the authors stated it focused on adults [50]. Table 2 summarizes the main findings of the qualitative studies. A broad range of themes emerged including knowledge of PA and its benefits as well as barriers and facilitators to participation among SA. All but one study discussed some aspect of these themes [54]. The study by Pollard & Guell explored the feasibility of using accelerometers with South Asian women and how well they were able to recall PA from the previous week in order to determine the appropriateness of using questionnaires with the group [54].

**Table 2:** Summary of qualitative studies focusing on South Asian women and physical activity

**Author, publication date& quality rating**	**Country**	**aim**	**Methods sample**	**Sample**	**Main themes**
Darr et al., 2008 -Strong+	UK	To examine and compare illness beliefs of South Asian and European patients with CHD about lifestyle changes	In-depth interviews	N(Pakistani/Muslim men) = 10	Perceptions: Vigorous PA* seen as unnecessary, just keep mobile to achieve adequate PA levels
				N(Pakistani/Muslim women) = 10	Barriers: Lack of time and uncomfortable walking alone
				N(Indian/Sikh men) = 7	
				N(Indian/Sikh women) = 5	
				N(Indian/Hindu men) = 9	
				N(Indian/Hindu men) = 9	
				N(Indian/Hindu women) = 4	
				N(European men) = 10	
				N(European women) = 10	
				Age range: 40-83	
Galadas et al., 2012 - Strong	Canada	To describe Punkabi Sikh patients’ perceived barriers to engaging in physical exercise following myocardial infarction (MI)	Semi-structured interviews	N(Punjab men) = 10	Perceptions: Difficulty determining safe PA levels
				N(Punjab women) = 5	Informal exercise versus structured PA in a gym would be better
				Age range: 48-80	Social networks disrupted after migrating to Canada and therefore difficult to make friends with whom to do PA with
					Barriers: Fatigue and weakness after MI
Grace et al., 2008 -Strong	UK	To understand lay beliefs and attitudes, religious teachings, and professional perceptions in relation to diabetes prevention in the Bangladeshi community	Focus groups for lay SA religious leaders	N(lay SA men) = 37	Perceptions: ‘Namaz’ is term used to refer to exercise
				N(lay SA women) = 43	PA is seen as way to care for the body and for controlling weight
				N(Religious leader men) = 14	Walking best form of activity to maintain modesty
				N(lay religious leader women) = 15	PA central to Muslim way of life
				Mean age: 35 +/-2 standard deviations	
Horne et al., 2009 -Weak/moderate	UK	To identify salient beliefs that influence uptake and adherence to exercise for fall prevention among community dwelling Caucasian and SA 60-70 years old	Ethnographic participant observation, focus groups, and semi structured interviews	FG: N(White men) = 14	Perceptions: PA not considered necessary if a person is healthy
				N(White women) = 44	Barriers: Limited knowledge of PA and its benefits
				N(SA men) = 16	Unaware of benefits of PA such as balance and improved mobility
				N(SA women) = 13	Fear of injury if participate in PA Lack of confidence to do PA
				Interviews:	
				N(White men) = 9	
				N(White women) = 14	
				N(SA men) = 7	
				N(SA women) = 10	
				Mean age range: 65.2-66.1	
Kalra et al., 2004 -Strong/moderate	US	To gather information on the perceptions of cardiovascular risk within the Asian Indian community and to identify opportunities to design health promotion and intervention programs	Focus groups	N = 57 Asian Indian men and women	Perceptions: Urban dwellers more likely to want to do PA in a gym
				FG size and sex unspecified	Rural dwellers knew to walk and caretaking was PA
				Ages unspecified	
Lawton et al., 2006 -Strong	UK	To explore perceptions and experiences of undertaking physical activity as part of diabetes care	In-depth interviews	N(SA) = 32	Perceptions: Should do PA
				N(Pakistani men) = 11	Encouraged by health professional to walk
				N(Pakistani women) = 11	Barriers: Lack of time, fear to go out alone, no culturally sensitive facilities, domestic duties take priority over PA
				N(SA Indian men) = 4	
				N(SA Indian women) = 5	
				Age range:40s-70s	
Mohan et al., 2008 - Moderate	Australia	To report lifestyle factors of Asian Indians in Australia in relation to CHD and explore factors that could inform health education and cardiac rehabilitation programs in achieving lifestyle behavior changes	Semi-structured interviews	N = 8	Barriers: Family is a higher priority than PA; loneliness and lack of support after migration
				N(SA Indian men) = 5	
				N(SA Indian women) = 3	
				6 born in India, 2 born in Fiji	
				Age range: 41-80	
Pollard & Guell, 2011 -Moderate	UK	To explore the facility and confidence with which women were able to recall information on PA, as required by questionnaires	Semi-structured interviews, 24-hour PA recall and accelerometry	N = 22 (British Pakistani women only)	Recall of PA: Women unlikely to accurately quantify time or intensities of daily PA
				Age range: 24-61	Commonly used questionnaires unlikely to accurately capture PA levels
Sriskantharajah & Kai,2006 -Strong	UK	To explore influences on, and attitudes towards, physical activity among SA women with CHD and diabetes to inform secondary prevention strategies	Semi-structured interviews	N = 15 (women only)	Barriers: Uncertainty of what activities to do
				N(SA Indian) = 5	Selfish to take PA
				N(Pakistani) = 4	Language difficulties
				N(Bangladeshi) = 1	Modesty an issue
				N(East African Asian) = 2	
				N(Sri Lankan) = 3	
				Age range:26- + 70	
Walseth 2008 -Moderate	Norway	To explore social network dimension of social capital, and whether participation in sport leads to accumulation of social capital for young women with an immigrant background	In-depth interviews	N = 15 (women only from Pakistan, Turkey, Morocco, Iran, Syria, Gambia, and Kosovo)	Perceptions: Sport clubs strengthened established friendships
				Age range: 16-25	Focus on similarities among each other rather than differences

+Quality rating from checklist from Critical Skills Appraisal Programme(8) in which the assessor ranks a series of questions from weak to strong based on their assessment of rigor, credibility and relevance of the study to answering each question. *Physical activity; **South Asian; *** Focus group.

### Knowledge of physical activity and its benefits

Nine studies highlighted participants’ knowledge of PA and its benefits [23,41,46,48-51,53,58]. In three studies respondents reported awareness that they should be participating in regular PA and that it has some general health benefits [23,50,58]. In the study by Horne et al., participants reported not being aware of the health benefits of PA [49]. Although there was a general awareness, five studies reported that there was confusion as to what types and how much PA to perform as well as confusion about specific health benefits [46,48,49,53,58].

### Barriers to participating in physical activity

Nine studies reported barriers to PA participation among SA [23,46,48-53,58]. Major barriers were those due to cultural differences with the dominant society and structural barriers. Five studies reported that SA as well as their families and communities would view taking time out to participate in PA as a selfish act [23,48,51,53,58]. Women reported that in SA culture, a woman’s focus is meant to be on the family and she should perform domestic duties over all other activities [23,53]. Those women who participated in PA or wanted to, were concerned about the stigma that might come from others in their community [23,48,58]. Moreover they reported that caretaking duties left them with no time for PA [23,46,58]. Five studies cited culturally inappropriate facilities as a barrier to PA participation in this population [46,48,52,53,58]. Examples included mixed-sex facilities such as swimming pools that do not consider the women’s requirement for modesty, and the use of male instructors [23,48,58]. Four studies found that women were less likely to participate in PA outside their home if they had difficulties speaking English, the language of the wider society [23,48,52,53]. The concept of fatalism or the idea that health is Allah’s will was expressed in two studies [48,49].

Structural barriers such as fear for personal safety were cited in five studies [23,46,49,52,53]. Many women were worried for their safety if they were to go out into the neighborhood unaccompanied [23,46], while others were fearful of exacerbating an illness or disability by doing too much PA or becoming too tired while out in the neighborhood alone [46,49,53]. Three studies cited poor weather as the main barrier for low PA participation [23,46,52]. Finally, lack of time [23,46,58], money [48], and access to open spaces [46] were additional structural barriers noted.

### Facilitators to participating in physical activity

Facilitators to PA participation emerged in four studies [23,48,49,53]. A common facilitator seen in all studies was motivation to participate in PA as a way to care for the health of the body and to prevent or alleviate illness and disease [23,48,49,53]. Two studies offered solutions to lack of PA motivation in SA [23,48]. Having exercise equipment in the home was seen as one way to motivate people to be physically active and eliminate several barriers to participation [23]. Education about Muslim faith was also seen as a way to motivate the South Asian community since PA was seen as central to the Muslim way of life [48].

### Final synthesis & discussion

The quantitative studies included in this review broadly indicate that SA’s PA levels are lower than the general or white population of their host countries, though SA Indian women generally had higher levels when compared to other SA women (Bangladeshi and Pakistani women) of the host countries [4,10,19,20,41]. There were no randomized controlled trials available for inclusion in this review, which may indicate that there is not enough high quality evidence on PA or ST in this population from which to draw conclusions. Self-report surveys were used to measure PA participation in all but two studies [22,32]; it is recognised these measures have limitations such as recall bias and misinterpretation of questions [14,59]. Eight studies reported using questions on PA from a validated questionnaire, though these have been validated on white populations, not SA populations [25,27,28,34,36,37,39,42]. The study that used an accelerometer to measure PA levels chose to use a device that is often used in conjunction with a heart rate monitor and is typically used to measure the energy expenditure (in kcals) of PA [32]. The findings that women of SA origin perform little PA based on their energy expenditure are similar to other studies that have used the *Caltrac* to assess PA in other ethnic minority women such as African American women in the US [60].

This device is known to overestimate energy expenditure of activities such as walking and running and therefore may not be the best choice of accelerometer [56]. Other accelerometers that are commonly used are the Actigraph and the ActivePAL [44]. These can be worn without the use of a heart rate monitor and are widely used to measure the intensity and duration of PA [44]. More high quality studies with rigorous study designs and methods are needed to assess levels of PA and ST in this population.

Heterogeneity within SA groups based on country of origin/birth and diversity of socioeconomic status (SES), religious beliefs and cultural practices make insights from these studies difficult to generalize and should be interpreted with caution [61,62]. Of the 26 quantitative studies reviewed only 13 included any information on SES and a range of markers of SES were measured across studies [4,8,19-21,28-30,33,34,36,38,39,42]. Measures of SES included household income [4,8,21,34,36,42], education level [4,8,19,20,28,30,33], and deprivation indexes such as Index of Multiple Deprivation scores [37] and the Townsend Deprivation Index [4]. Other potential mediating or confounding factors such as employment status [18,28], disease status [4,22,32,38,39,41], religion [19,36], stress levels [36] and racial discrimination [20,36] were reported and controlled for in physical activity analyses, although these were not collected in all studies.

Conclusions from these studies are based on the limited and often missing information on sampling, methods and findings obtained from papers. Most studies gave minimal or no information on the translation of surveys into the appropriate languages. As the qualitative studies reviewed here have indicated, English language skills are limited in this group and efforts should be made by researchers to make the translation and interview process more transparent. Measurement and definition of PA varied widely, making it difficult to quantify activity across all studies. Moreover terms such as PA, vigorous, moderate, exercise, etc. have different meanings in the English language, making them challenging to understand by those whose first language is not English [63]. As evidenced by this review, ST is largely overlooked within the current research on SA. Only two studies reported ST and this was self-reported data [36,38]. Based on the evidence indicating low levels of PA, this population is likely to have high rates of ST and sedentary behaviors. Future research is needed to assess levels of ST and contextualize sedentary behaviors since these have been identified as important independent risk factors for CVD. Additionally, it is difficult to compare PA across studies since the domain of PA measured, and the methods used to assess PA, varied across studies. The majority of quantitative studies measured LTPA. It may be the case that SA women do not engage in LTPA but do engage in more household or occupational activities. There may be considerable amounts of PA that are not being captured by the currently published studies, therefore more investigation is needed into the type of PA that SA women currently engage in.

The qualitative studies reviewed for this paper varied by methods, sample size, and research focus. While most studies reported that they chose themes or interview questions a priori, there was little description of how these were chosen. Mays & Pope (2003) recommend using the presence of a theoretical framework as one aspect of assessing quality of qualitative studies [15]. However only three studies in this review [15,47,51] identified and reported the theoretical framework that underpinned the study.

Knowledge of PA and its benefits was found to be lacking among SA. Horne et al. (2009) found that there was disagreement on the understanding of the difference between exercise and PA [49]. This misunderstanding can be compounded by health practitioners or health promotion professionals giving only general recommendations, and failing to provide detail on what activities should be performed, at what intensity and for what duration [53]. Awareness of specific health benefits of PA such as falls prevention, cardiovascular health, and stress relief was low among many SA [23,49,52,58]. Of those respondents who knew about the benefits of PA and had knowledge of which activities to participate in, few reported that they were successful in actually performing any activity [23,46,49,58] or did not believe that they needed PA for their own health [46,49,52,58]. In contrast, Kalra et al. (2004) found that most respondents (who were all of SA Indian descent) did do some PA [50].

While the majority of the qualitative studies addressed barriers and facilitator to PA, there was little agreement in the findings. There are several possible reasons for this. The term “South Asian” is used to group together people from the India, Pakistan, Bangladesh, and Sri Lanka, but this grouping is one of convenience and fails to address the differences that may be found between these diverse countries and cultures. The studies were also guided by different research goals. For example, some focused on PA in SA with type 2 diabetes [23,48] or heart disease [47,51-53,58], and others on falls prevention for older SA [49]. Finally, the method of data collection, i.e. individual interviews vs. focus groups, may provide information on individual’s thoughts and perceptions (interviews) or reveal the thoughts and perceptions of the collective (focus groups) [61]. Some common themes across these two methods were identified. These included barriers to PA due to differences of the host culture and the SA culture, structural barriers such as neighborhood safety, and facilitators such as the use of faith and education to encourage activity.

Put in the context of global PA and ST among ethnic minority groups, the findings from this review are consistent with those of many previous studies on other ethnic minority groups. Examples include studies on African Americans and Hispanics in the United States where researchers have found similar barriers to those of the SA reviewed here, including cultural barriers such as proper dress for PA and lack of time, money and facilities for PA [1,57,64]. Studies on Muslim minority groups have also found comparable results to these, referring to an incongruence of the minority culture and the host population as a main barrier to PA behaviors [65]. Future research should focus on improved methods of working within minority cultures to reduce barriers to PA and discourage ST. While there are similarities in the barriers to PA, the solutions to low PA among these groups may be varied due to the specific characteristics of individuals and communities. These should therefore be explored further and employed to promote increased levels of PA and reduce ST among SA.

This review has built a detailed description of all the evidence published since 1980 on SA and PA from both quantitative and qualitative sources. This method of synthesizing both types of studies has some limitations. There is currently no standardized way of extracting data or assessing the quality of both types of studies [61], therefore more than one method was used. This analysis suffers from a lack of detail from some papers on the sample, methods, and findings. Publication and researcher bias are also a consideration, although efforts were made to procure all studies, published or unpublished, and to use multiple researchers in the identification of studies, data extraction and assessment of quality. Finally, due to a lack of randomized controlled trials and significant heterogeneity between studies, a meta-analysis of results was not possible.

Although not a traditional systematic review, this review has closely followed the rigorous standards of conducting systematic reviews. The strengths of this review include transparent methodology and the inclusion of all types of research to produce the most comprehensive narrative evidence on PA and SA. This review updates and improves upon previous work through integration of study types to provide the most comprehensive picture of existing evidence in this area [63,66]. This systematic synthesis of studies also allows for the development of more informed recommendations for future research needs and intervention strategies.

### Implications

These findings have implications for researchers, policy specialists and health practitioners. It is clear from the findings of this review that there is little standardization of PA measurement or terminology used to describe SA groups across studies. This reduces the ability to compare findings across studies and to make any useful generalizations. It is recommended that researchers use objective measures of PA (e.g., accelerometers) more widely among ethnic minority groups. Only two of the 26 quantitative studies [20,22] in this review used objective measurements and only one of those used accelerometers [32]. While there are other objective measurements that might be considered to be the gold standard, accelerometers offer the ability to accurately and consistently monitor PA and avoid recall bias, translation issues, and have a high degree of reliability over time [22,59]. The wider use of this technology will help to standardize PA measures and allow for better comparisons across studies. While objective measurement is recommended, it is not always a practical option. Therefore when self-report methods must be used in lieu of objective measurement, it is recommended that researchers used the IPAQ in order to standardize measurement across studies [67]. The IPAQ can be translated into a range of languages and dialects and used for a wide variety of participants, [38,59,67]. Additionally, this review illustrates the need for more theory-based research to better understand the social, structural, economic, and cultural factors contributing to the PA and ST in this group. Moreover, there is currently not enough data to ascertain whether there is a consistent difference between women of various SA subgroups, and if there is, what that difference might be.

Policy specialists should consider the alarmingly low rates of PA among SA and press for more evidence-based interventions and programs aimed at this population. The results reported here suggest that healthcare and health promotion practitioners need to provide more detailed guidance on the type, frequency and duration of PA for their patients and clients in this population. There is also concern among SA that participating in PA will aggravate current disease or illness and practitioners should promote education on the benefits of PA even for those with disease. The use of translators and leaders in the faith communities may assist in disseminating these messages.

## Conclusions

This review has identified the quantitative and qualitative studies on PA among SA. All quantitative studies showed a trend toward low activity levels in this group, though outcome measures varied widely. Although findings were limited, they suggest that barriers to PA may be overcome by working within cultural norms and to provide more education on the safety and benefit of PA. The evidence base for these conclusions is limited and it is recommended that more research be done in this area to close gaps in knowledge and provide the information needed to develop and disseminate culturally appropriate interventions that increase PA and reduce ST in this population.

## Competing interests

Both Authors declare that they have no competing interests.

## Authors’ contributions

WSB designed the study and collected the data. WSB and JLT assessed and analyzed the data. WSB drafted the manuscript and JLT critically revised subsequent versions of the paper. Both authors read and approved the final version of the paper.
